# Changes in sex difference in swimming speed in finalists at FINA World Championships and the Olympic Games from 1992 to 2013

**DOI:** 10.1186/2052-1847-6-25

**Published:** 2014-06-25

**Authors:** Stefanie Wild, Christoph Alexander Rüst, Thomas Rosemann, Beat Knechtle

**Affiliations:** 1 Institute of General Practice and for Health Services Research, University of Zurich, Zurich, Switzerland; 2 Gesundheitszentrum St. Gallen, Vadianstrasse 26, 9001 St. Gallen, Switzerland

**Keywords:** Swimming speed, Sex difference

## Abstract

**Background:**

This study investigated swimming speeds and sex differences of finalists competing at the Olympic Games (*i.e.* 624 female and 672 male athletes) and FINA World Championships (*i.e.* 990 women and 1008 men) between 1992 and 2013.

**Methods:**

Linear, non-linear and multi-level regression models were used to investigate changes in swimming speeds and sex differences for champions and finalists.

**Results:**

Regarding finalists in FINA World Championships and Olympic Games, swimming speed increased linearly in both women and men in all disciplines and race distances. Male world champions’ swimming speed remained stable in 200 m butterfly, 400 m, 800 m and 1,500 m freestyle. Considering women, swimming speed remained unchanged in 50 m and 400 m freestyle. In the Olympic Games, swimming speed of male champions remained unchanged in 200 m breaststroke, 50 m, 400 m, 800 m and 1,500 m freestyle. Female Olympic champions’ swimming speed remained stable in 100 m and 200 m backstroke, 100 m butterfly, 200 m individual medley, 50 m and 200 m freestyle. Evaluating sex differences between finalists in FINA World Championships, results showed a linear decrease in 100 m breaststroke and 200 m butterfly and a non-linear increase in 100 m backstroke. In finals at the Olympic Games, the sex difference decreased linearly for 100 m backstroke, 400 m and 800 m freestyle. However, a linear increase for 200 m butterfly can be reported. Considering Olympic and world champions, the sex difference remained stable in all disciplines and race distances.

**Conclusion:**

Swimming speed of the finalists at the Olympic Games and FINA World Championships increased linearly. The top annual female swimmers increased swimming speed rather at longer race distances (*i.e.* 800 m and 1,500 m freestyle, 200 m butterfly, and 400 m individual medley), whereas the top annual male swimmers increased it rather at shorter race distances (*i.e.* 100 m and 200 m freestyle, 100 m butterfly, and 100 m breaststroke). Sex difference in swimming was unchanged in Olympic and world champions. Finalists and champions at the Olympic Games and FINA World Championships reduced the sex difference with increasing race distance.

## Background

In recent years, there have been numerous studies investigating swimming speed trends in different Olympic disciplines [1-3]. Berthelot *et al.*[1] analyzed 3,263 swimming world records established for all quantifiable official contests since the first Olympic Games. They distinguished an increase in swimming speed until the 1970s, where a plateau in swimming speed was reached. It was hypothesized that the maximum possible physiological sports performance for human species will be reached in one generation, which implies that half of all world records will not be improved more than 0.05% by 2027 [1]. Nevill *et al.*[2] investigated whether swimming world records were beginning to plateau in 100 m, 200 m and 400 m freestyle swimming. They reported a similar plateau effect between the 1980s and 1990s. Notwithstanding, swimming speed increased again by roughly 2% at the beginning of the 21^st^ century [4]. This unforeseen swimming speed improvement was investigated and reported by several studies [4-6].

Smith *et al.*[6] analyzed on scientific tools used in physiological and psychological disciplines. They concluded that athletes could be distinguished on the basis of their psychological skills and emotional competencies. Colwin [5] investigated on training processes and how they improved across the years. It was summarized that in any country the future starts with inspired coaches and not the administrators or scientists. Beside physiological, psychological characteristics as well as trainings processes, Berthelot *et al.*[4] focused on material science in swimming by measuring the impact of the three successive generations of swimsuits on human performance. As outcome of this study three bursts of swimming speed improvements were reported occurring in 2000, 2008 and 2009.

Summarizing the findings from Colwin [5], Smith *et al.*[6] and Berthelot *et al.*[4], mainly four factors were identified to explain the unforeseen swimming speed improvements at the beginning of the 21^st^ century: First, the better evaluation of swimmers by physiological parameters, psychological skills, and emotional competencies [6], second, more efficient training processes based on better training control [5], third, deeper pools and more effective ‘antiwave’ lane ropes [5] and, fourth, new drag-reducing swimsuits [4].

Considering differences between female and male elite athletes, swimming speed remained stable between 1957 and 2006. Interestingly, female athletes improved their swimming speed in 100 m, 200 m and 400 m freestyle faster than their male counterparts during the 1960s and 1970s, but never outperformed their male counterparts [2]. Between 1991 and 1995, Tanaka *et al.*[7] reported a decrease in sex difference with increasing race distance. In detail, for freestyle swimmers, the sex difference in swimming speed decreased from 19 ± 1% for 50 m to 11 ± 1% for 1,500 m. Despite this development, sex differences between the top-six finalists at each FINA World Championship and the Olympic Games in 100 m freestyle and 100 m backstroke swimming increased again between 2000 and 2005 [8]. This led to an overall increase in sex difference between 1981 and 2006. Buhl *et al.*[9] compared medley and freestyle swimming speeds for national (*i.e.* top ten elite Swiss athletes) and international swimmers (*i.e.* top eight FINA World Championship athletes) between 1994 and 2011. These authors reported that the sex difference for national and international athletes in 400 m medley as well as freestyle was lower compared to the 200 m distances. Wolfrum *et al.*[10] focused on national and international breaststroke and freestyle disciplines and reported a decrease in the sex difference with increasing race distance. Relating to sex difference, Rüst *et al.*[11] also reported a decrease in sex difference with increasing race distance from 50 m to 800 m amongst Swiss elite freestyle swimmers ranked on the Swiss high score list between 2006 and 2010. However, they reported that for 1,500 m freestyle, the sex difference increased compared to 800 m freestyle. Buhl *et al.*[9] compared medley and freestyle swimming speeds for national (*i.e.* top ten elite Swiss athletes) and international swimmers (*i.e.* top eight FINA World Championship athletes) between 1994 and 2011. For both, national and international athletes, the sex difference decreased with increasing race distance in both individual medley and freestyle [9].

Beside studies on the swimming speed and sex difference trends in swimming speed at indoor pool competitions, also open-water long-distance races were investigated for the same purpose [12-18]. Vogt *et al.*[12] studied the sex difference of elite open-water swimmers competing in 10 km swimming competitions at European Championships, FINA World Championships, World Cup races and the Olympic Games between 2008 and 2012. The study’s outcomes showed that the swimming speed remained stable for the best elite female and male athletes. Even compared to other long-distance races (*e.g.* ultra-running, ultra-cycling) the sex difference in 10 km swimming with 7% was remarkably low. Eichenberger *et al.*[14] analysed the 26.4 km ‘Marathon Swim’ held in Lake Zurich, Switzerland. During the last decade, the sex difference remained stable at ~11.5%. The same authors analyzed the ‘Zurich 12 h Swim’ between 1996 and 2010, whereby results showed that the annual best swimming speed was not significantly different between male and female athletes [13]. A similar event, namely the 46 km ‘Manhattan Island Marathon Swim’ was analyzed for sex difference in swimming speed [15]. The observed time period was from 1983 to 2013. As result these authors stated that the best women were approximately 12-14% faster than the best men. Rüst *et al.* investigated on the 36 km ‘Maratona del Golfo Capri-Napoli’ [16] and the 32 km ‘Traversée internationale du lac St-Jean’ event [17] regarding sex difference trends in swimming speed. For the ‘Maratona del Gofo Capri-Napoli’ and the ‘Traversée international du lac St-Jean’ the time period observed was from 1954 to 2013 and from 1955 to 2012, respectively. In conclusion, the fastest women reduced the gap with the fastest men at the 36 km ‘Maratona del Golfo Capri-Napoli’ linearly from ~40% to ~5.6% [16] whereas the sex difference in swimming speed remained unchanged at 8.8 ± 5.6% at the 32 km ‘Traversée internationale du lac St-Jean’ competition [17]. Zingg *et al.*[18] investigated on sex difference trends in swimming speed for elite male and female swimmers competing in 5 km, 10 km and 25 km open-water FINA World Cup races held between 2000 and 2012. These authors reported that the sex difference in swimming remained stable in 5 km, decreased linearly in 10 km and increased linearly in 25 km.

To the best of our knowledge, no studies investigated swimming speed and sex difference trends in swimming speed in all swimming disciplines held at FINA World Championships and Olympic Games for very recent years. So far, studies have only focused on swimming speed and sex difference trends in swimming speed in a limited range of disciplines and race distances. The present study is the first to focus on a complete evaluation of swimming speed with a focus on sex difference in swimming speed of all indoor swimming events held at FINA World Championships and the Olympic Games.

Therefore, the aim of the present study was to examine the trends in swimming speed and sex difference in swimming speed in all swimming disciplines held at FINA World Championships and the Olympic Games between 1992 and 2013. We hypothesized (*i*) an improvement in swimming speeds in all disciplines across the years, (*ii*) a stability of sex differences in swimming speed over the period from 1992 to 2013, and (*iii*) a decrease in sex difference in swimming speed with increasing race distance.

## Methods

### Ethics

All procedures used in the study were approved by the Institutional Review Board of the Canton of St. Gallen, Switzerland. A waiver of the requirement for informed consent of the participants was granted given the fact that the study involved the analysis of publicly available data.

### Data sampling and data analysis

In this study, swimmers competing in the finals of FINA World Championships as well as the Olympic Games between 1992 and 2013 were analyzed and compared regarding change in swimming speed and sex difference. All data was obtained from the publicly accessible FINA and OMEGA web sites [19,20]. Only data from the finals were used in order to assure that athletes were only included once per discipline and respective year. The data set used for analysis is based on information from the European Swimming Federation (LEN) rankings database and the results and ranking database from the Belgium, Canadian, Dutch, Faroe, Polish, Portuguese, Slovakian and Swiss Swimming Federation [21]. Since data before 1992 were incomplete, no race results of competitions held before 1992 were included in the analysis. Additionally, only those race distances were considered which are contested by both women and men.

In total, data was available for 990 women and 1,008 men at FINA World Championships and for 624 women and 672 men at the Olympic Games. Those data originated from 15 finals for each discipline, race distance and sex. The only exception was female individuals in 1,500 m freestyle where only 7 finals were held during that time period. Prior to analysis, race times were converted to swimming speed (m/s) using the equation [swimming speed in m/s] = [race distance in m]/[race time in s]. Changes in swimming speed of the winners and all eight finalists were analyzed for every competition, race distance, swim style, age and sex. Additionally, the sex difference between men and women was calculated and analyzed as follows. Sex differences in swimming speed were determined using the equation ([women swimming speed] – [men swimming speed])/[men swimming speed] × 100, for pairs of equally placed athletes (*e.g.*, men’s and women’s 1^st^ place speeds, men’s and women’s 2^nd^ place speeds, etc*.*). The mean and standard deviation were then calculated for all pairs. To facilitate reading, all sex differences were transformed to absolute values before analyzing.

### Statistical analysis

Prior to statistical analysis, each data set was tested for normal distribution using D’Agostino and Pearson omnibus normality test as well as for homogeneity of variances using Levene’s Test. Single and multi-level regression analysis investigated changes in swimming speed and age of the finalists. A hierarchical regression model was used to avoid the impact of a cluster-effect on results where a particular athlete from a specific country competed more than once in one year. Regression analyses of swimming speed were corrected for age of athletes to prevent a misinterpretation of the ‘age-effect’ as a ‘time-effect’. Since the change in sex difference in endurance is assumed to be non-linear [22], we additionally calculated the non-linear regression model that fits the data best. When the best-fit model was not a linear but a non-linear polynomial regression, we compared the best-fit non-linear model to the linear model using Akaike’s Information Criteria (AIC) and F-test in order to show which model would be the most appropriate to explain the trend of the data. Statistical analyses were performed using IBM SPSS Statistics (Version 21, IBM SPSS, Chicago, IL, USA) and GraphPad Prism (Version 6.01, GraphPad Software, La Jolla, CA, USA). Significance was accepted at *p <* 0.05 (two-tailed for *t*-tests). Results were reported in the text and figures as mean ± standard deviation (SD).

## Results

### Multiple participations

**Figure 1:**
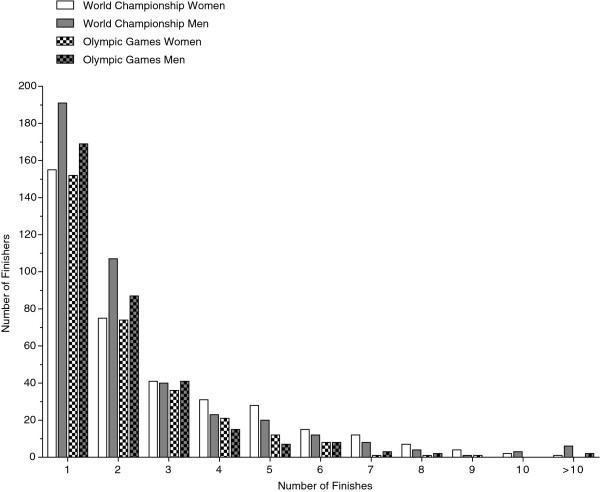
Statistical overview across single athletes who participated multiple times in the Olympic Games and FINA World Championships.

In total, data was analysed for 306 female and 334 male individuals at the Olympic Games and 371 female and 415 male individuals at FINA World Championships. In the Olympic Games, 152 women and 169 men participated only once, 74 women and 87 men participated twice. Regarding FINA World Championships, 155 women and 191 men competed once, 75 women and 107 men participated twice. One woman and six men competed in FINA World Championships more than ten times. In the Olympic Games, no women participated more than nine times, whereas there were two men which participated more than ten times (Figure 1).

### Changes in swimming speeds over the years in the FINA World Championships

**Figure 2:**
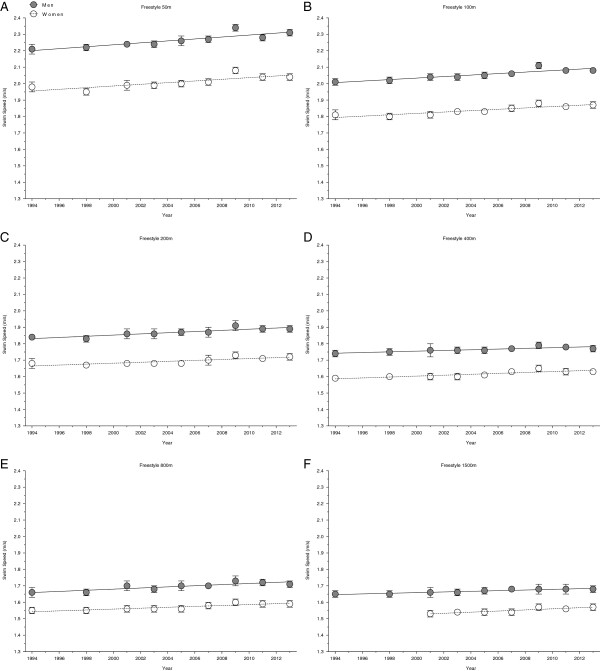
Changes in swimming speed for the finalists in 50 m (Panel A), 100 m (Panel B), 200 m (Panel C), 400 m (Panel D), 800 m (Panel E) and 1,500 m (Panel F) freestyle at FINA World Championships between the years 1994 and 2013.

**Figure 3:**
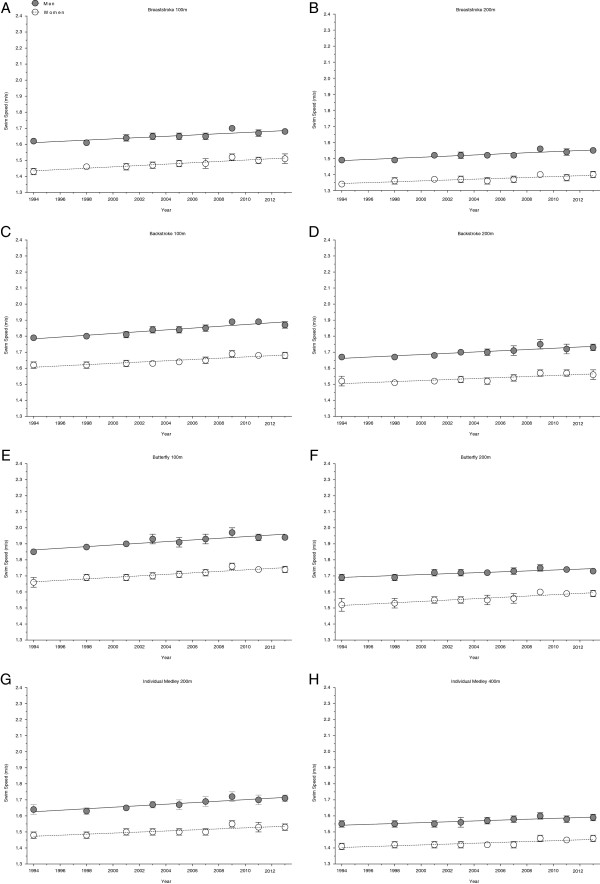
Changes in swimming speed for the finalists in 100 m breaststroke (Panel A), 200 m breaststroke (Panel B), 100 m backstroke (Panel C), 200 m backstroke (Panel D), 100 m butterfly (Panel E), 200 m butterfly (Panel F), 200 m individual medley (Panel G) and 400 m individual medley (Panel H) at FINA World Championships between the years 1994 and 2013.

**Figure 4:**
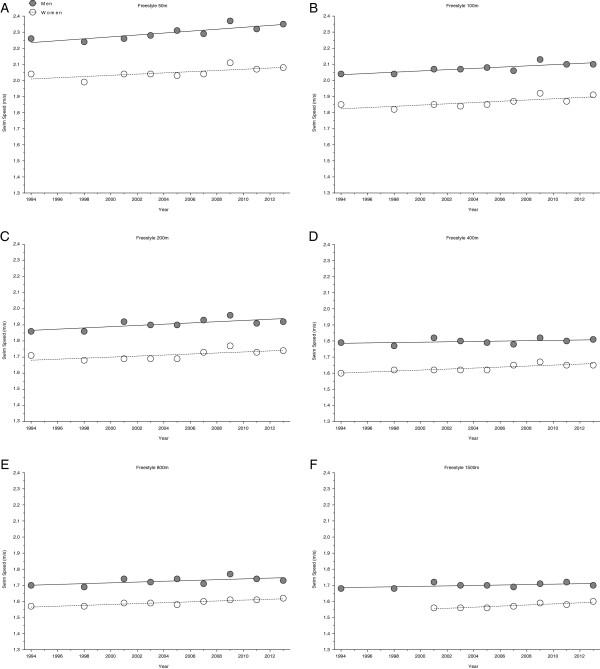
Changes in swimming speed for the champions in 50 m (Panel A), 100 m (Panel B), 200 m (Panel C), 400 m (Panel D), 800 m (Panel E) and 1,500 m (Panel F) freestyle at FINA World Championships between the years 1994 and 2013.

**Figure 5:**
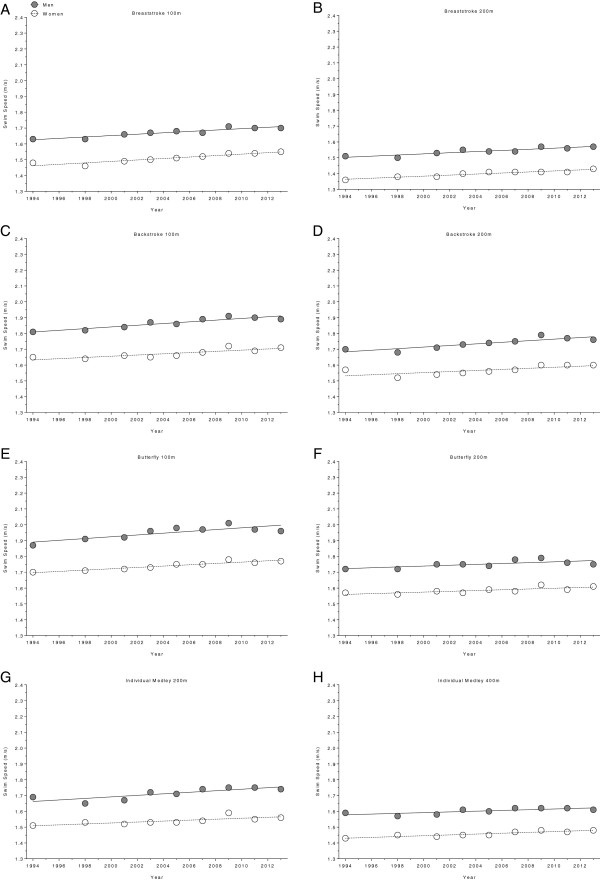
Changes in swimming speed for the champions in 100 m breaststroke (Panel A), 200 m breaststroke (Panel B), 100 m backstroke (Panel C), 200 m backstroke (Panel D), 100 m butterfly (Panel E), 200 m butterfly (Panel F), 200 m individual medley (Panel G) and 400 m individual medley (Panel H) at FINA World Championships between the years 1994 and 2013.

Figures 2 and 3 show the changes in swimming speed for the finalists in the FINA World Championships. In detail, the results for 50 m, 100 m, 200 m, 400 m, 800 m and 1500 m freestyle are presented in Figure 2 whereas the results for 100 m and 200 m breaststroke, 100 m and 200 m backstroke, 100 m and 200 m butterfly as well as 200 m and 400 m individual medley are reflected in Figure 3. Women’s and men’s swimming speeds increased both linearly for all disciplines and race distances over time (Table 1). In Table 2, the swimming speeds in 1994 and 2013 for both, female and male, finalists at FINA World Championships are presented. Overall, the swimming speeds in each discipline increased between 1994 and 2013 in both women and men. In Figures 4 and 5, changes in swimming speed for the world champions between 1994 and 2013 are depicted. Figure 4 focuses on the results for 50 m, 100 m, 200 m, 400 m, 800 m and 1500 m freestyle whereas Figure 5 shows the results for 100 m and 200 m breaststroke, 100 m and 200 m backstroke, 100 m and 200 m butterfly as well as 200 m and 400 m individual medley. In contrast to the finalists, no significant change in swimming speed for both, female and male world champions could be detected in the investigated disciplines (Table 3). For men, swimming speed remained stable in 200 m butterfly, 400 m freestyle, 800 m freestyle and 1,500 m freestyle at 1.75 ± 0.02 m/s, 1.8 ± 0.02 m/s, 1.72 ± 0.02 m/s and 1.7 ± 0.02 m/s, respectively. For female world champions, swimming speed remained unchanged in 50 m freestyle and 400 m freestyle at 2.05 ± 0.03 m/s and at 1.63 ± 0.02 m/s, respectively. However, in 200 m backstroke, female world champions’ swimming speed increased non-linearly, whereas the speed of their male counterparts increased linearly. This non-linear increase was best described with a polynomial 4^th^ degree. In the remaining disciplines swimming speeds of female and male world champions increased linearly over time (Table 3).

**Table 1:** Multi-level regression analysis for changes in swimming speed for the finalists in the FINA World Championships in freestyle, breaststroke, backstroke, butterfly and individual medley with correction for multiple participations and age of the athletes

**Stroke**	**Sex**	**Distance (m)**	***β***	**SE ( *β *)**	**Stand. *β***	**T**	***p***
Freestyle	Male	50	0.006	0.001	0.812	10.487	< 0.001
		100	0.005	0.000	0.803	11.396	< 0.001
		200	0.004	0.001	0.675	7.528	< 0.001
		400	0.002	0.000	0.496	4.719	< 0.001
		800	0.004	0.001	0.651	6.958	< 0.001
		1500	0.002	0.000	0.503	4.714	< 0.001
	Female	50	0.005	0.001	0.698	8.175	< 0.001
		100	0.004	0.001	0.749	9.154	< 0.001
		200	0.003	0.000	0.689	7.828	< 0.001
		400	0.003	0.000	0.731	8.480	< 0.001
		800	0.002	0.000	0.590	5.925	< 0.001
		1500	0.003	0.001	0.544	4.601	< 0.001
Breaststroke	Male	100	0.003	0.000	0.814	11.718	< 0.001
		200	0.003	0.000	0.816	11.719	< 0.001
	Women	100	0.005	0.000	0.816	11.719	< 0.001
		200	0.003	0.000	0.684	7.247	< 0.001
Backstroke	Male	100	0.005	0.000	0.847	12.895	< 0.001
		200	0.004	0.000	0.704	8.243	< 0.001
	Women	100	0.005	0.000	0.797	10.525	< 0.001
		200	0.003	0.000	0.684	7.247	< 0.001
Butterfly	Male	100	0.005	0.001	0.803	10.281	< 0.001
		200	0.003	0.000	0.641	6.845	< 0.001
	Women	100	0.005	0.000	0.814	11.074	< 0.001
		200	0.004	0.001	0.707	7.997	< 0.001
Individual medley	Male	200	0.005	0.001	0.749	9.189	< 0.001
		400	0.003	0.000	0.561	5.633	< 0.001
	Women	200	0.004	0.000	0.700	7.810	< 0.001
		400	0.003	0.000	0.657	7.110	< 0.001

**Table 2:** Change in swimming speed over time for the finalists in the FINA World Championships for women and men

**Swim speed (m · s**^**−1**^**)**				
**Sex**	**1994**		**2013**	
**50 m freestyle**				
Women	1.98 ± 0.03		2.04 ± 0.02	*
Men	2.21 ± 0.03		2.31 ± 0.02	*
**100 m freestyle**				
Women	1.81 ± 0.03		1.87 ± 0.02	*
Men	2.01 ± 0.02		2.08 ± 0.01	*
**200 m freestyle**				
Women	1.68 ± 0.03		1.72 ± 0.02	*
Men	1.84 ± 0.01		1.89 ± 0.02	*
**400 m freestyle**				
Women	1.59 ± 0.01		1.63 ± 0.01	*
Men	1.74 ± 0.02		1.77 ± 0.02	*
**800 m freestyle**				
Women	1.55 ± 0.02		1.59 ± 0.02	*
Men	1.66 ± 0.03		1.71 ± 0.02	*
**1,500 m freestyle**				
Women	1.53 ± 0.02	(2001)	1.57 ± 0.02	*
Men	1.65 ± 0.02		1.68 ± 0.02	*
**100 m breaststroke**				
Women	1.43 ± 0.02		1.51 ± 0.03	*
Men	1.62 ± 0.01		1.68 ± 0.01	*
**200 m breaststroke**				
Women	1.34 ± 0.01		1.40 ± 0.02	*
Men	1.49 ± 0.01		1.55 ± 0.01	*
**100 m backstroke**				
Women	1.62 ± 0.02		1.68 ± 0.02	*
Men	1.79 ± 0.01		1.87 ± 0.02	*
**200 m backstroke**				
Women	1.52 ± 0.03		1.56 ± 0.03	*
Men	1.67 ± 0.01		1.73 ± 0.02	*
**100 m butterfly**				
Women	1.66 ± 0.03		1.74 ± 0.02	*
Men	1.85 ± 0.01		1.94 ± 0.01	*
**200 m butterfly**				
Women	1.52 ± 0.04		1.59 ± 0.02	*
Men	1.69 ± 0.02		1.73 ± 0.01	*
**200 m individual medley**				
Women	1.48 ± 0.02		1.53 ± 0.02	*
Men	1.64 ± 0.03		1.71 ± 0.02	*
**400 m individual medley**				
Women	1.41 ± 0.02		1.46 ± 0.02	*
Men	1.55 ± 0.02		1.59 ± 0.02	*

Note: *p < 0.05.

**Table 3:** Multi-level regression analysis for change in swimming speed for the world champions in freestyle, breaststroke, backstroke, butterfly and individual medley with correction for multiple participations and age of the athletes

**Stroke**	**Sex**	**Distance (m)**	***β***	**SE ( *β *)**	**Stand. *β***	**T**	***p***
Freestyle	Male	50	0.006	0.001	0.846	3.997	0.007
		100	0.004	0.001	0.768	3.293	0.017
		200	0.004	0.002	0.846	2.797	0.310
		400	0.001	0.001	0.259	0.672	0.527
		800	0.002	0.001	0.593	1.754	0.130
		1500	0.001	0.001	0.514	1.478	0.190
	Female	50	0.004	0.002	0.693	2.206	0.070
		100	0.004	0.001	0.755	2.747	0.033
		200	0.003	0.001	0.694	2.844	0.029
		400	0.005	0.002	1.282	2.335	0.058
		800	0.003	0.000	0.985	7.017	< 0.001
		1500	0.003	0.001	0.906	4.285	0.013
Breaststroke	Male	100	0.005	0.001	1.126	6.187	0.001
		200	0.004	0.000	0.926	9.188	< 0.001
	Women	100	0.005	0.001	0.943	7.108	< 0.001
		200	0.004	0.000	0.926	9.188	< 0.001
Backstroke	Male	100	0.006	0.001	1.003	8.373	< 0.001
		200	0.005	0.001	0.854	3.346	0.016
	Women	100	0.004	0.001	0.800	3.970	0.007
		200	0.003	0.001	0.730	2.646	0.038
Butterfly	Male	100	0.006	0.002	0.827	3.590	0.011
		200	0.003	0.001	0.687	2.049	0.086
	Women	100	0.004	0.000	0.905	9.760	< 0.001
		200	0.003	0.001	0.687	2.049	0.086
Individual medley	Male	200	0.006	0.001	1.037	4.527	0.004
		400	0.002	0.001	0.761	2.697	0.036
	Women	200	0.003	0.001	0.754	2.930	0.026
		400	0.003	0.001	0.902	4.589	0.004

### Changes in swimming speeds over the years in the Olympic Games

**Figure 6:**
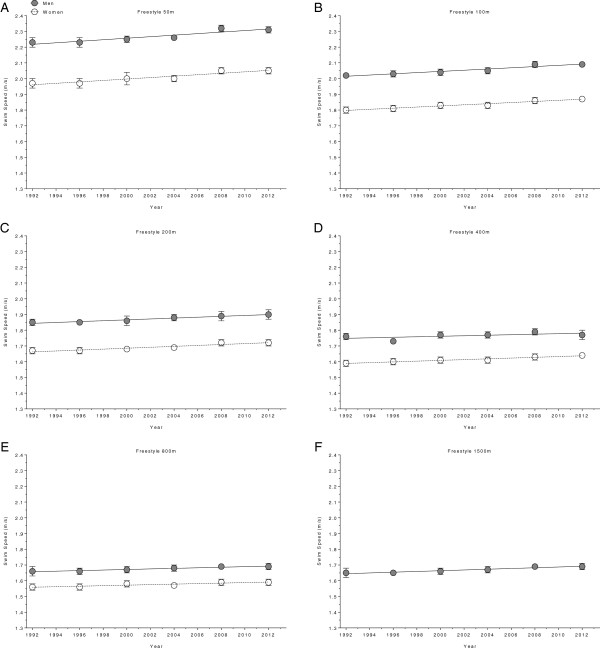
Changes in swimming speed for the finalists in 50 m (Panel A), 100 m (Panel B), 200 m (Panel C), 400 m (Panel D), 800 m (Panel E) and 1,500 m (Panel F) freestyle at the Olympic Games between the years 1992 and 2012.

**Figure 7:**
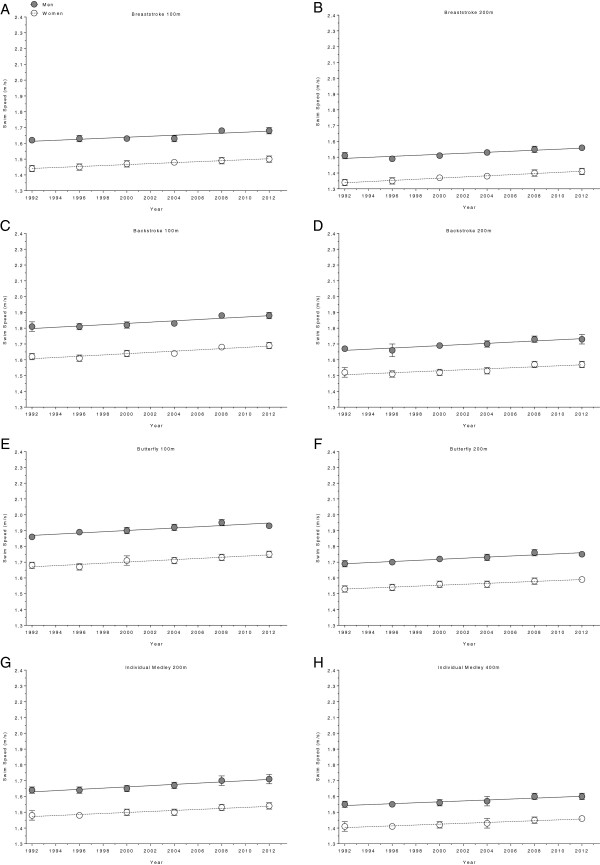
Changes in swimming speed for the finalists in 100 m breaststroke (Panel A), 200 m breaststroke (Panel B), 100 m backstroke (Panel C), 200 m backstroke (Panel D), 100 m butterfly (Panel E), 200 m butterfly (Panel F), 200 m individual medley (Panel G) and 400 m individual medley (Panel H) at the Olympic Games between the years 1992 and 2012.

**Figure 8:**
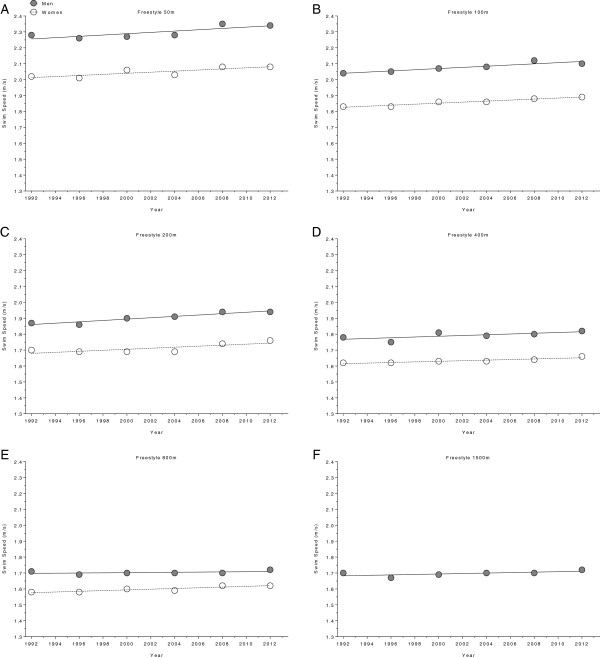
Changes in swimming speed for the champions in 50 m (Panel A), 100 m (Panel B), 200 m (Panel C), 400 m (Panel D), 800 m (Panel E) and 1,500 m (Panel F) freestyle at the Olympic Games between the years 1992 and 2012.

**Figure 9:**
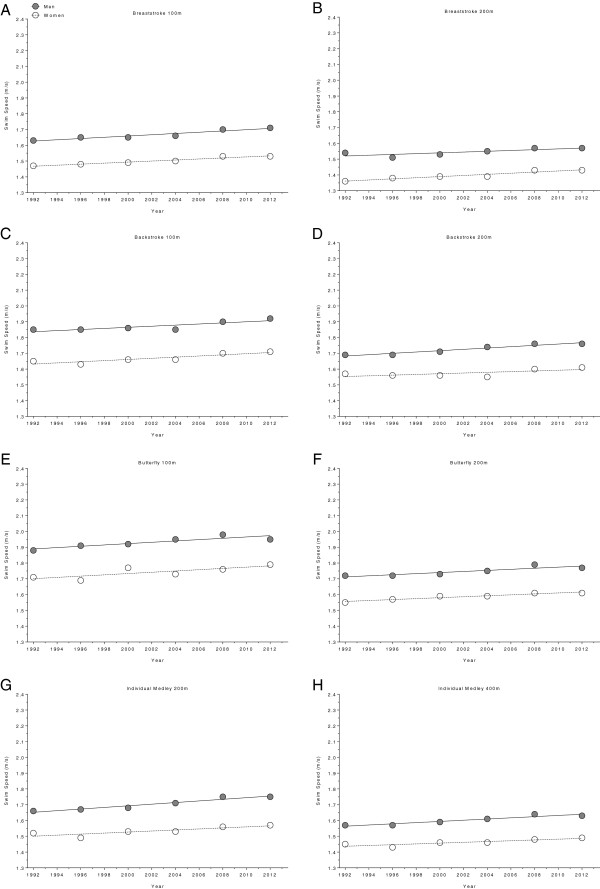
Changes in swimming speed for the champions in 100 m breaststroke (Panel A), 200 m breaststroke (Panel B), 100 m backstroke (Panel C), 200 m backstroke (Panel D), 100 m butterfly (Panel E), 200 m butterfly (Panel F), 200 m individual medley (Panel G) and 400 m individual medley (Panel H) at the Olympic Games between the years 1992 and 2012.

Similar to the FINA World Championships, swimming speed increased significantly and linearly for female and male finalists in the Olympic Games between 1992 and 2012 (Table 4). Further, Figures 6 and 7 represent the swimming speed over the years for 50 m freestyle, 100 m freestyle, 200 m freestyle, 400 m freestyle, 800 m freestyle, 1500 m freestyle and 100 m breaststroke, 200 m breaststroke, 100 m backstroke, 200 m backstroke, 100 m butterfly, 200 m butterfly, 200 m individual medley, 400 m individual medley, respectively. The corresponding numbers to these disciplines and distances are listed in Table 5. A comparison solely between Olympic champions revealed a slightly different result. Women’s swimming speed remained stable in 100 m backstroke with 1.67 ± 0.03 m/s, 200 m backstroke with 1.58 ± 0.02 m/s, 100 m butterfly with 1.74 ± 0.04 m/s, 200 m individual medley with 1.53 ± 0.03 m/s, 50 m freestyle with 2.05 ± 0.03 m/s and 200 m freestyle with 1.71 ± 0.03 m/s, respectively (Table 6). For men, swimming speed remained unchanged in 200 m breaststroke with 1.55 ± 0.02 m/s, 50 m freestyle with 2.3 ± 0.04 m/s, 400 m freestyle with 1.79 ± 0.02 m/s, 800 m freestyle with 1.7 ± 0.01 m/s and 1,500 m freestyle with 1.7 ± 0.02 m/s (Table 6). In the remaining disciplines, female and male Olympic champions’ swimming speed increased linearly over time, when corrected for multiple participation and age of athletes (Table 6). Figures 8 and 9 show these swimming speed trends in the time period between 1992 and 2012. In detail, the results for 50 m, 100 m, 200 m, 400 m, 800 m and 1500 m freestyle are depicted in Figure 8 whereas Figure 9 shows the results for 100 m and 200 m breaststroke, 100 m and 200 m backstroke, 100 m and 200 m butterfly as well as 200 m and 400 m individual medley.

**Table 4:** Multi-level regression analysis for changes in swimming speed for finalists in the Olympic Games in freestyle, breaststroke, backstroke, butterfly and individual medley with correction for multiple participations and age of the athletes

**Stroke**	**Sex**	**Distance (m)**	***β***	**SE ( *β *)**	**Stand. *β***	**T**	***p***
Freestyle	Male	50	0.005	0.001	0.856	9.415	< 0.001
		100	0.004	0.000	0.841	10.247	< 0.001
		200	0.003	0.000	0.671	6.030	< 0.001
		400	0.002	0.000	0.530	4.087	< 0.001
		800	0.002	0.000	0.537	4.245	< 0.001
		1500	0.002	0.000	0.623	5.282	< 0.001
	Female	50	0.004	0.001	0.718	6.840	< 0.001
		100	0.003	0.000	0.800	8.683	< 0.001
		200	0.003	0.000	0.785	8.254	< 0.001
		400	0.002	0.000	0.703	6.790	< 0.001
		800	0.002	0.000	0.620	5.832	< 0.001
		1500	-	-	-	-	-
Breaststroke	Male	100	0.003	0.000	0.779	7.649	< 0.001
		200	0.003	0.000	0.801	8.939	< 0.001
	Women	100	0.003	0.000	0.799	8.689	< 0.001
		200	0.003	0.000	0.889	10.386	< 0.001
Backstroke	Male	100	0.004	0.000	0.763	8.171	< 0.001
		200	0.004	0.001	0.711	6.761	< 0.001
	Women	100	0.004	0.000	0.823	9.141	< 0.001
		200	0.003	0.001	0.662	5.902	< 0.001
Butterfly	Male	100	0.004	0.000	0.863	10.782	< 0.001
		200	0.004	0.000	0.866	10.489	< 0.001
	Women	100	0.004	0.000	0.740	7.850	< 0.001
		200	0.003	0.000	0.731	7.083	< 0.001
Individual medley	Male	200	0.003	0.000	0.733	7.130	< 0.001
		400	0.003	0.001	0.624	5.447	< 0.001
	Women	200	0.004	0.001	0.779	7.723	< 0.001
		400	0.003	0.000	0.674	6.199	< 0.001

**Table 5:** Change in swimming speed over time for the finalists in the Olympic Games for women and men

**Swim speed (m · s**^**−1**^**)**			
**Sex**	**1992**	**2012**	
**50 m freestyle**			
Women	1.97 ± 0.03	2.05 ± 0.02	*
Men	2.23 ± 0.03	2.3 ± 10.02	*
**100 m freestyle**			
Women	1.80 ± 0.02	1.87 ± 0.01	*
Men	2.02 ± 0.01	2.09 ± 0.01	*
**200 m freestyle**			
Women	1.67 ± 0.02	1.72 ± 0.02	*
Men	1.85 ± 0.02	1.90 ± 0.03	*
**400 m freestyle**			
Women	1.59 ± 0.02	1.64 ± 0.01	*
Men	1.76 ± 0.02	1.77 ± 0.03	*
**800 m freestyle**			
Women	1.56 ± 0.02	1.59 ± 0.02	*
Men	1.66 ± 0.03	1.69 ± 0.02	*
**1,500 m freestyle**			
Women	-	-	
Men	1.65 ± 0.03	1.69 ± 0.02	*
**100 m breaststroke**			
Women	1.44 ± 0.02	1.50 ± 0.02	*
Men	1.62 ± 0.01	1.68 ± 0.02	*
**200 m breaststroke**			
Women	1.34 ± 0.02	1.41 ± 0.02	*
Men	1.51 ± 0.02	1.56 ± 0.01	*
**100 m backstroke**			
Women	1.62 ± 0.02	1.69 ± 0.02	*
Men	1.81 ± 0.03	1.88 ± 0.02	*
**200 m backstroke**			
Women	1.52 ± 0.03	1.57 ± 0.02	*
Men	1.67 ± 0.01	1.73 ± 0.03	*
**100 m butterfly**			
Women	1.68 ± 0.02	1.75 ± 0.02	*
Men	1.86 ± 0.01	1.93 ± 0.01	*
**200 m butterfly**			
Women	1.53 ± 0.02	1.59 ± 0.01	*
Men	1.69 ± 0.02	1.75 ± 0.01	*
**200 m individual medley**			
Women	1.48 ± 0.03	1.54 ± 0.02	*
Men	1.64 ± 0.02	1.71 ± 0.03	*
**400 m individual medley**			
Women	1.41 ± 0.03	1.46 ± 0.01	*
Men	1.55 ± 0.02	1.60 ± 0.02	*

Note: *p < 0.05.

**Table 6:** Multi-level regression analysis for change in swimming speed for the Olympic Champions in freestyle, breaststroke, backstroke, butterfly and individual medley with correction for multiple participations and age of the athletes

**Stroke**	**Sex**	**Distance (m)**	***β***	**SE ( *β *)**	**Stand. *β***	**T**	***p***
Freestyle	Male	50	0.004	0.001	0.826	2.862	0.064
		100	0.004	0.001	0.909	3.496	0.040
		200	0.004	0.001	0.923	4.636	0.019
		400	0.003	0.001	0.847	2.710	0.073
		800	0.001	0.001	0.483	1.083	0.358
		1500	0.002	0.001	0.898	2.561	0.083
	Female	50	0.004	0.001	0.884	2.822	0.067
		100	0.003	0.000	0.914	8.164	0.004
		200	0.003	0.001	0.737	2.468	0.090
		400	0.002	0.000	1.010	4.756	0.018
		800	0.002	0.001	0.891	3.354	0.044
		1500	-	-	-	-	-
Breaststroke	Male	100	0.003	0.001	0.758	5.260	0.013
		200	0.003	0.001	0.798	2.410	0.095
	Women	100	0.003	0.000	1.003	8.998	0.003
		200	0.004	0.001	1.053	3.885	0.030
Backstroke	Male	100	0.003	0.001	0.769	4.159	0.025
		200	0.004	0.001	0.961	6.540	0.007
	Women	100	0.003	0.001	0.839	2.966	0.059
		200	0.002	0.001	0.684	1.604	0.207
Butterfly	Male	100	0.004	0.001	0.844	4.513	0.020
		200	0.004	0.001	1.030	3.669	0.035
	Women	100	0.004	0.002	0.778	2.057	0.132
		200	0.003	0.001	0.958	5.700	0.011
Individual medley	Male	200	0.005	0.001	0.956	6.424	0.008
		400	0.004	0.001	0.962	5.072	0.015
	Women	200	0.002	0.001	0.500	2.025	0.136
		400	0.002	0.000	0.696	6.724	0.007

### Changes in sex differences over the years in FINA World Championships

**Figure 10:**
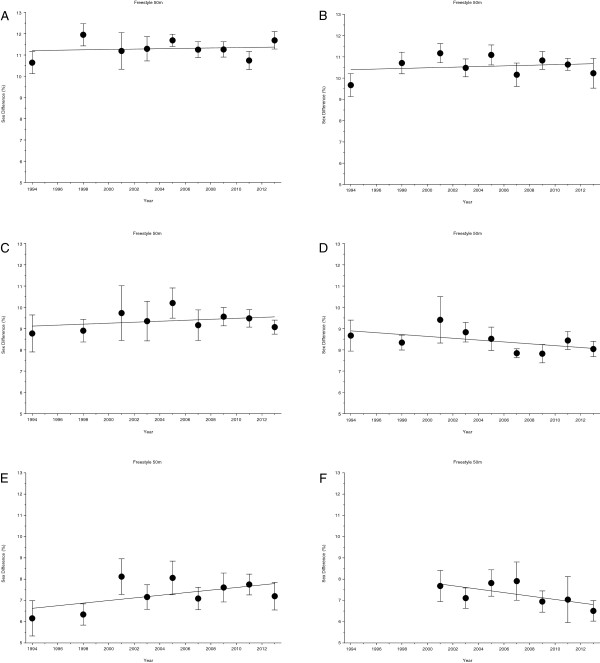
Changes in sex difference for the finalists in 50 m (Panel A), 100 m (Panel B), 200 m (Panel C), 400 m (Panel D), 800 m (Panel E), and 1500 m (Panel F) freestyle at FINA World Championships between the years 1994 and 2013.

**Figure 11:**
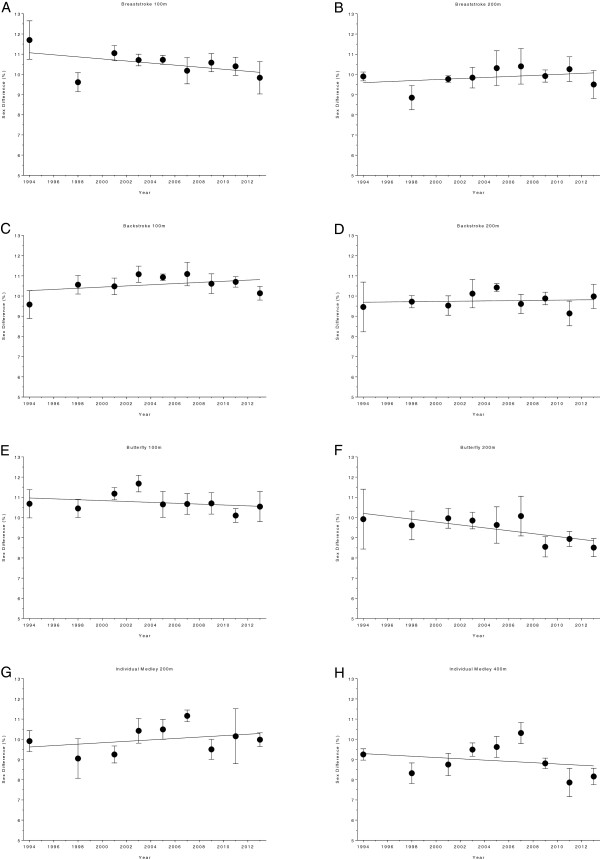
Changes in sex difference for the finalists in 100 m breaststroke (Panel A), 200 m breaststroke (Panel B), 100 m backstroke (Panel C), 200 m backstroke (Panel D), 100 m butterfly (Panel E), 200 m butterfly (Panel F), 200 m individual medley (Panel G) and 400 m individual medley (Panel H) at FINA World Championships between the years 1994 and 2013.

**Figure 12:**
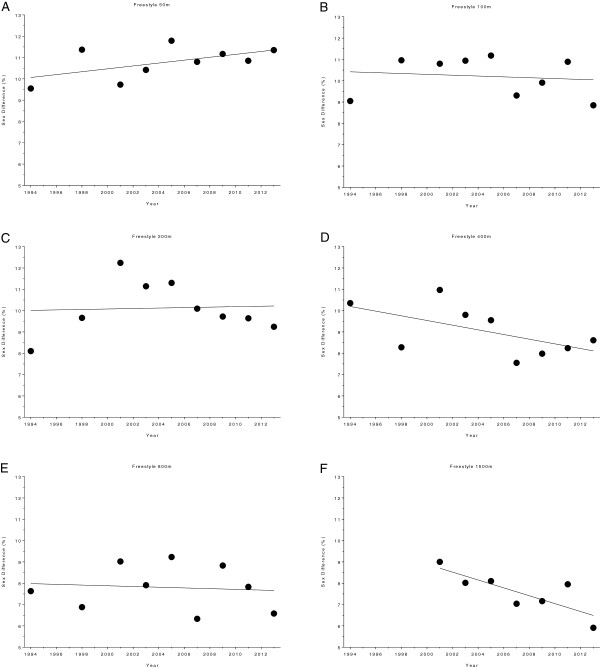
Changes in sex difference for the champions in 50 m (Panel A), 100 m (Panel B), 200 m (Panel C), 400 m (Panel D), 800 m (Panel E), and 1500 m (Panel F) freestyle at FINA World Championships between the years 1994 and 2013.

**Figure 13:**
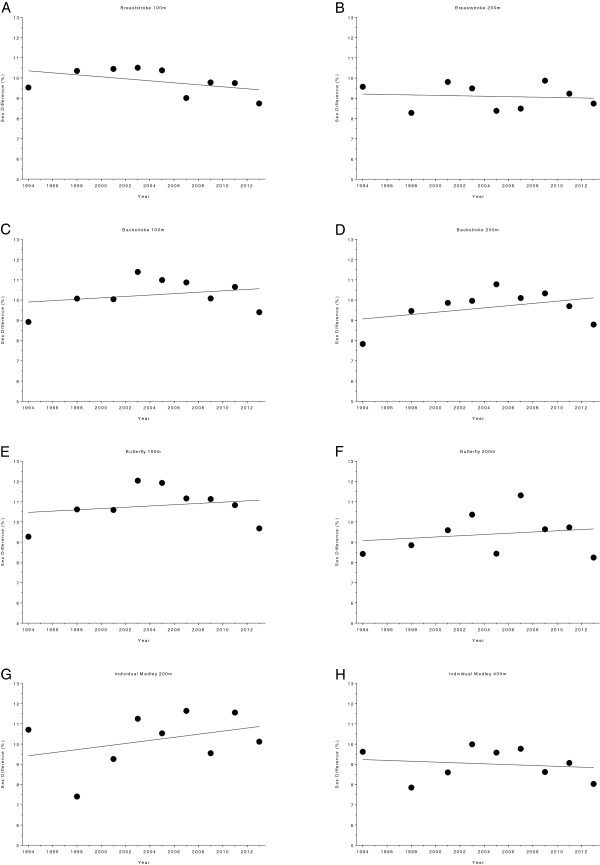
Changes in sex difference for the champions in 100 m breaststroke (Panel A), 200 m breaststroke (Panel B), 100 m backstroke (Panel C), 200 m backstroke (Panel D), 100 m butterfly (Panel E), 200 m butterfly (Panel F), 200 m individual medley (Panel G) and 400 m individual medley (Panel H) at FINA World Championships between the years 1994 and 2013.

Figures 10 and 11 show the changes in sex difference for the finalists in FINA World Championships held between 1994 and 2013. The distribution of the results for the various disciplines and distances is as follows. Results for 50 m, 100 m, 200 m, 400 m and 800 m freestyle are depicted in Figure 10. Results for 100 m breaststroke, 200 m breaststroke, 100 m backstroke, 200 m backstroke, 100 m butterfly, 200 m butterfly, 200 m individual medley and 400 m individual medley are shown in Figure 11. In 100 m breaststroke and 200 m butterfly the sex differences decreased linearly over the years (Table 7). The corresponding numbers are 11.7 ± 0.95% to 9.83 ± 0.8% and 9.92 ± 1.48% to 8.51 ± 0.45%, respectively (Table 8). In 100 m backstroke, a non-linear increase (*i.e.* polynomial 2^nd^ degree) from 9.58 ± 0.69% to 10.14 ± 0.34% can be reported. For all other disciplines, the sex difference remained stable (Table 7). In Figure 12 (*i.e.* 50 m, 100 m, 200 m, 400 m and 800 m freestyle) and Figure 13 (*i.e.* 100 m breaststroke, 200 m breaststroke, 100 m backstroke, 200 m backstroke, 100 m butterfly, 200 m butterfly, 200 m individual medley and 400 m individual medley), changes in sex difference for the world champions between 1994 and 2013 are depicted. In contrast to the finalists, no significant change in sex difference for both, female and male world champions, could be detected in any of the investigated disciplines (Table 9).

**Table 7:** Multi-level regression analysis for changes in sex difference in swimming speed for finalists in the FINA World Championships in freestyle, breaststroke, backstroke, butterfly and individual medley with correction for multiple participations

	***β***	**SE ( *β *)**	**Stand. *β***	**T**	***p***
50 m freestyle	0.005	0.048	0.056	0.112	0.916
100 m freestyle	0.016	0.020	0.362	0.777	0.481
200 m freestyle	0.023	0.063	0.181	0.367	0.732
400 m freestyle	0.007	0.052	0.071	0.142	0.894
800 m freestyle	−0.090	0.045	−0.705	−1.988	0.118
100 m breaststroke	−0.051	0.015	−0.375	−3.384	0.001
200 m breaststroke	0.025	0.014	0.209	1.785	0.079
100 m backstroke	0.029	0.012	0.271	2.356	0.021
200 m backstroke	0.006	0.014	0.055	0.460	0.647
100 m butterfly	−0.003	0.021	−0.025	−0.168	0.867
200 m butterfly	0.028	0.012	0.325	2.333	0.024
200 m individual medley	0.035	0.018	0.230	1.978	0.052
400 m individual medley	−0.033	0.017	−0.221	−1.884	0.064

**Table 8:** Change in sex difference over time for the finalists in the FINA World Championships

**Sex difference (%)**			
**1994**		**2013**	
**50 m freestyle**			
10.64 ± 0.52		11.69 ± 0.41	
**100 m freestyle**			
9.67 ± 0.54		10.23 ± 0.70	
**200 m freestyle**			
8.77 ± 0.87		9.07 ± .033	
**400 m freestyle**			
8.67 ± 0.73		8.04 ± 0.36	
**800 m freestyle**			
6.16 ± 0.83		7.20 ± 0.65	
**1,500 m freestyle**			
7.68 ± 0.73	(2001)	6.51 ± 0.48	
**100 m breaststroke**			
11.70 ± 0.95		9.83 ± 0.80	*
**200 m breaststroke**			
9.90 ± 0.22		9.50 ± 0.69	
**100 m backstroke**			
9.58 ± 0.69		10.14 ± 0.34	*
**200 m backstrocke**			
9.46 ± 1.23		9.98 ± .060	
**100 m butterfly**			
10.68 ± 0.70		10.54 ± 0.74	
**200 m butterfly**			
9.92 ± 1.48		8.51 ± 0.45	*
**200 m individual medley**			
9.91 ± 0.52		9.98 ± 0.34	
**400 m individual medley**			
9.25 ± 0.28		8.16 ± 0.41	

Note: *p < 0.05.

**Table 9:** Multi-level regression analysis for changes in sex difference in swimming speed for the world champions in freestyle, breaststroke, backstroke, butterfly and individual medley with correction for multiple participations

	***β***	**SE ( *β *)**	**Stand. *β***	**T**	***p***
50 m freestyle	0.068	0.038	0.555	1.767	0.121
100 m freestyle	−0.020	0.056	−0.132	−0.353	0.735
200 m freestyle	0.011	0.076	0.054	0.144	0.889
400 m freestyle	−0.110	0.058	−0.580	−1.883	0.102
800 m freestyle	−0.017	0.065	−0.097	−0.259	0.803
100 m breaststroke	−0.049	0.035	−0.471	−1.413	0.200
200 m breaststroke	−0.011	0.038	−0.106	−0.281	0.787
100 m backstroke	0.035	0.046	0.272	0.749	0.478
200 m backstroke	0.055	0.049	0.388	1.113	0.302
100 m butterfly	0.032	0.055	0.214	0.579	0.581
200 m butterfly	0.031	0.061	0.186	0.501	0.632
200 m individual medley	0.076	0.077	0.351	0.993	0.354
400 m individual medley	−0.021	0.047	−0.166	−0.444	0.670

### Changes in sex differences over the years in the Olympic Games

**Figure 14:**
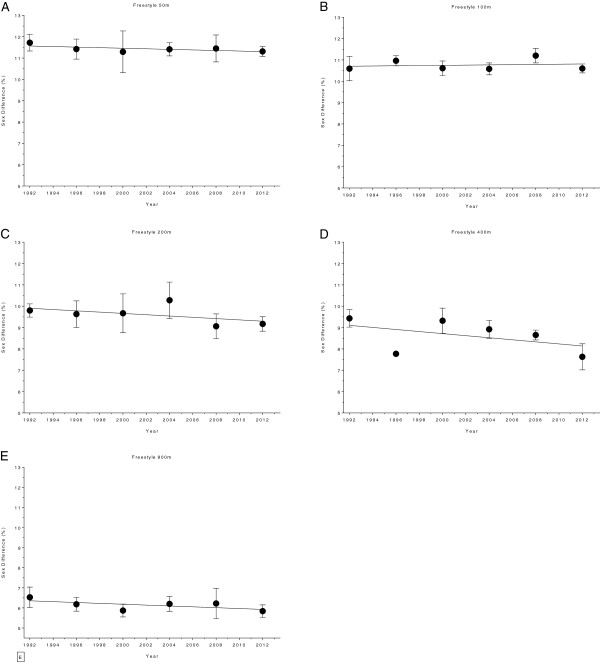
Changes in sex difference for the finalists in 50 m (Panel A), 100 m (Panel B), 200 m (Panel C), 400 m (Panel D) and 800 m (Panel E) freestyle at the Olympic Games between the years 1992 and 2012.

**Figure 15:**
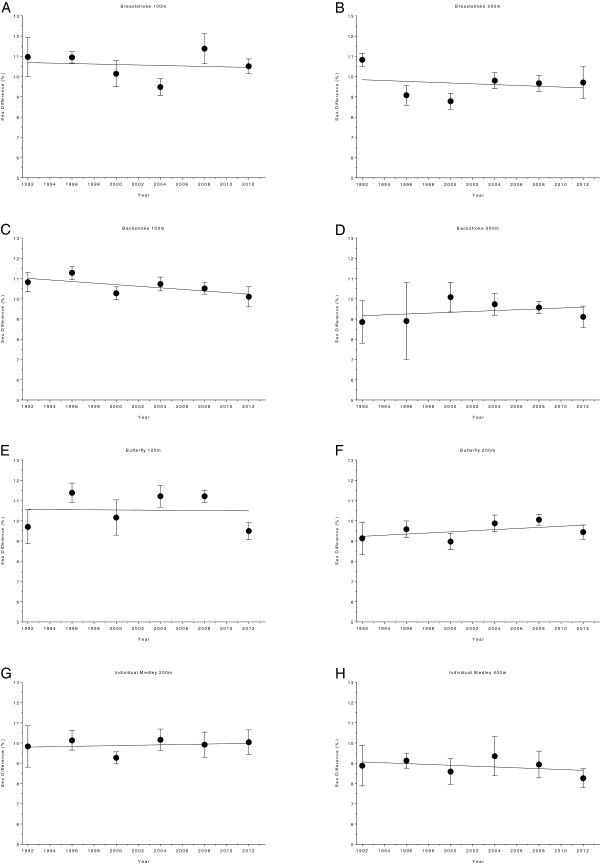
Changes in sex difference for the finalists in 100 m breaststroke (Panel A), 200 m breaststroke (Panel B), 100 m backstroke (Panel C), 200 m backstroke (Panel D), 100 m butterfly (Panel E), 200 m butterfly (Panel F), 200 m individual medley (Panel G) and 400 m individual medley (Panel H) at the Olympic Games between the years 1992 and 2012.

**Figure 16:**
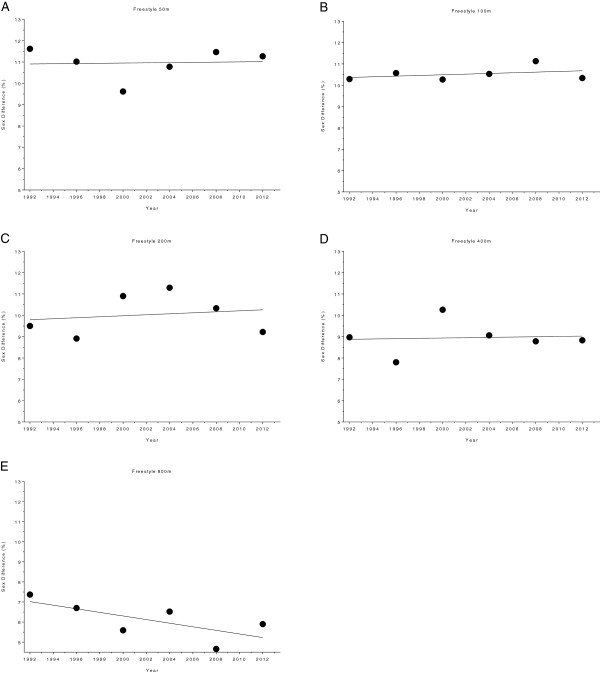
Changes in sex difference for the champions in 50 m (Panel A), 100 m (Panel B), 200 m (Panel C), 400 m (Panel D) and 800 m (Panel E) freestyle at the Olympic Games between the years 1992 and 2012.

**Figure 17:**
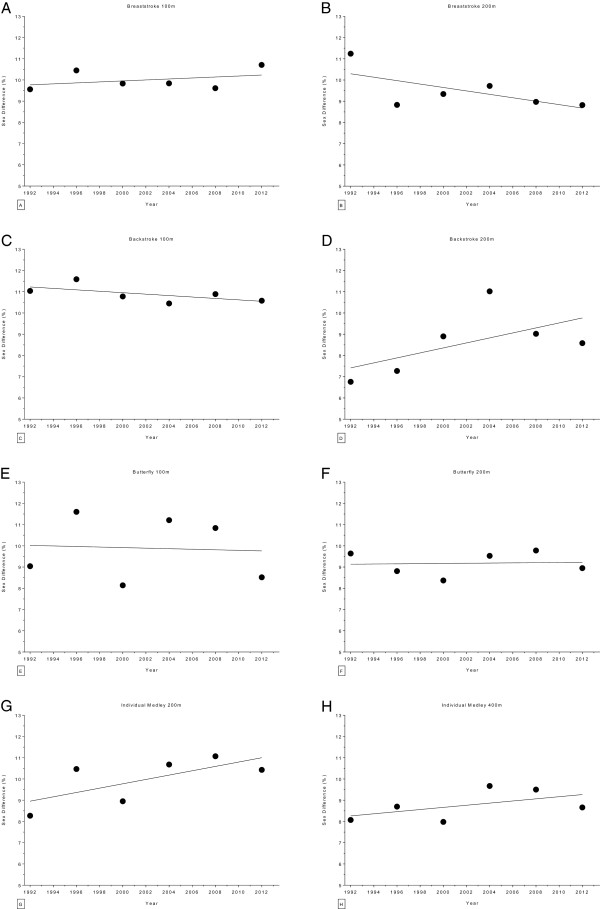
Changes in sex difference for the champions in 100 m breaststroke (Panel A), 200 m breaststroke (Panel B), 100 m backstroke (Panel C), 200 m backstroke (Panel D), 100 m butterfly (Panel E), 200 m butterfly (Panel F), 200 m individual medley (Panel G) and 400 m individual medley (Panel H) at the Olympic Games between the years 1992 and 2012.

The changes in sex difference for the finalists in the Olympic Games are given in Figure 14 (*i.e.* 50 m, 100 m, 200 m, 400 m and 800 m freestyle) and Figure 15 (*i.e.* 100 m breaststroke, 200 m breaststroke, 100 m backstroke, 200 m backstroke, 100 m butterfly, 200 m butterfly, 200 m individual medley and 400 m individual medley). In 100 m backstroke, 400 m freestyle and 800 m freestyle, the sex difference decreased linearly during the analyzed time period, from 10.82 ± 0.47% to 10.10 ± 0.5%, from 9.43 ± 0.41% to 7.63 ± 0.61% and from 6.53 ± 0.51% to 5.84 ± 0.31%, respectively (Table 10). However, a linear increase from 9.13 ± 0.8% to 9.44 ± 0.35% can be reported for 200 m butterfly (Table 11). Changes in sex difference for the world champions between 1992 and 2012 in 50 m, 100 m, 200 m, 400 m and 800 m freestyle are depicted in Figure 16 whereas Figure 17 shows the changes in sex difference for 100 m breaststroke, 200 m breaststroke, 100 m backstroke, 200 m backstroke, 100 m butterfly, 200 m butterfly, 200 m individual medley and 400 m individual medley. In contrast to the finalists, no significant change in sex difference for both, female and male Olympic Champions, could be detected in any of the investigated disciplines (Table 12).

**Table 10:** Multi-level regression analysis for changes in sex difference in swimming speed for finalists in the Olympic Games in freestyle, breaststroke, backstroke, butterfly and individual medley with correction for multiple participations

	***β***	**SE ( *β *)**	**Stand. *β***	**T**	***p***
50 m freestyle	−0.014	0.012	−0.170	−1.171	0.248
100 m freestyle	0.005	0.009	0.087	0.592	0.557
200 m freestyle	−0.030	0.015	−0.284	−2.010	0.050
400 m freestyle	−0.048	0.016	−0.409	−3.038	0.004
800 m freestyle	−0.021	0.010	−0.295	−2.098	0.041
100 m breaststroke	−0.012	0.018	−0.098	−0.668	0.507
200 m breaststroke	−0.020	0.017	−0.174	−1.198	0.237
100 m backstroke	−0.039	0.010	−0.507	−3.990	0.000
200 m backstroke	0.021	0.022	0.140	0.957	0.344
100 m butterfly	−0.003	0.021	−0.025	−0.168	0.867
200 m butterfly	0.028	0.012	0.325	2.333	0.024
200 m individual medley	0.009	0.014	0.095	0.644	0.523
400 m individual medley	−0.021	0.016	−0.187	−1.289	0.204

**Table 11:** Change in sex difference over time for the finalists in the Olympic Games

**Sex difference (%)**			
**1992**		**2012**	
	**50 m freestyle**		
11.72 ± 0.39	11.31 ± 0.23		
	**100 m freestyle**		
10.59 ± 0.57	10.60 ± 0.21		
	**200 m freestyle**		
9.80 ± 0.31	9.17 ± 0.34		
	**400 m freestyle**		
9.43 ± 0.41	7.63 ± 0.61		*
	**800 m freestyle**		
6.53 ± 0.51	5.84 ± 0.30		*
	**100 m breaststroke**		
10.97 ± 0.97	10.51 ± 0.36		
	**200 m breaststroke**		
10.83 ± 0.32	9.71 ± 0.79		
	**100 m backstroke**		
10.82 ± 0.47	10.10 ± 0.50		*
	**200 m backstroke**		
8.85 ± 1.05	9.11 ± 0.53		
	**100 m butterfly**		
9.70 ± 0.84	9.49 ± 0.42		
	**200 m butterfly**		
9.13 ± 0.80	9.44 ± 0.35		*
	**200 m individual medley**		
9.83 ± 1.02	10.04 ± 0.61		
	**400 m individual medley**		
8.88 ± 1.0	8.26 ± 0.47		

Note: *p < 0.05.

**Table 12:** Multi-level regression analysis for changes in sex difference in swimming speed for the Olympic champions in freestyle, breaststroke, backstroke, butterfly and individual medley with correction for multiple participations

	***β***	**SE ( *β *)**	**Stand. *β***	**T**	***p***
50 m freestyle	0.005	0.048	0.056	0.112	0.916
100 m freestyle	0.016	0.020	0.362	0.777	0.481
200 m freestyle	0.023	0.063	0.181	0.367	0.732
400 m freestyle	0.007	0.052	0.071	0.142	0.894
800 m freestyle	−0.090	0.045	−0.705	−1.988	0.118
100 m breaststroke	0.023	0.029	0.368	0.792	0.473
200 m breaststroke	−0.081	0.047	−0.652	−1.719	0.161
100 m backstroke	−0.034	0.021	−0.627	−1.608	0.183
200 m backstroke	0.118	0.081	0.586	1.447	0.221
100 m butterfly	−0.013	0.100	−0.065	−0.129	0.903
200 m butterfly	0.004	0.037	0.060	0.120	0.911
200 m individual medley	0.102	0.053	0.693	1.923	0.127
400 m individual medley	0.050	0.040	0.535	1.266	0.274

## Discussion

The aim of this study was to examine the trends in swimming speed and the changes in sex differences in all swimming disciplines held at the Olympic Games and FINA World Championships between 1992 and 2013. We hypothesized that (*i*) swimming speed would increase across the years, (*ii*) the sex difference would remain unchanged in the disciplines freestyle, breaststroke, backstroke, butterfly and individual medley across all race distances and (*iii*) the sex difference would decrease with increasing race distance. The main findings were (*i*) a linear increase in swimming speed of the overall top eight finalists at the Olympic Games and FINA World Championships, (*ii*) the fastest female swimmers increased their swimming speed rather at longer race distances (*i.e.* 800 m freestyle, 1,500 m freestyle, 200 m butterfly, 400 m individual medley), whereas the fastest male swimmers increased it rather at shorter race distances (*i.e.* 100 m freestyle, 200 m freestyle, 100 m butterfly, 100 m breaststroke), (*iii*) an unchanged sex difference in terms of swimming speed for Olympic and world champions, (*iv*) linear changes of sex differences concerning the swimming speed of all finalists, with the exception of a non-linear increase for the finalists in 100 m backstroke at FINA World Championships, and (*v*) for both, the finalists and champions at the Olympic Games and FINA World Championships, the sex difference was highest in the shortest race distances and lowest in the longest race distances.

### Increase in swimming speeds over the years

Between 1992 and 2013, the swimming speeds of the top eight women and men competing in the finals at the Olympic Games and FINA World Championships increased linearly. Several studies concerning performance evolution in swimming sports have been conducted over the last decade [4-6,12,23]. Factors explaining this development include technological progress such as more effective ‘antiwave’ lane ropes or new drag-reducing swim suits as well as the swimmer’s physiological and psychological skills due to better training conditions [4-6]. The present outcome supports these results. The fact that not even the beginning of a plateauing phase became apparent suggests that the observed linear improvement will continue in the next years. However, the time period analyzed might be too short to make long-term predictions regarding linear or non-linear trends. Nevertheless, our findings contradict the results of a preceding study which showed that swimming speeds reached a plateau phase beyond the 1970s [2].

Furthermore, the results differ from the reports in Vogt *et al.*[12], where performances of elite open-water swimmers at the 10 km FINA competitions from 2008 to 2012 were analyzed, and the performance of the top ten female swimmers remained stable, whereas it decreased for the top ten male swimmers during this period of time. Zingg *et al.*[23] analyzed the differences between 5 km, 10 km 25 km races from 2000 to 2012. They showed that swimming performance of the top ten female athletes improved in 10 km but decreased in 5 km and 25 km. The top ten male athletes’ swimming performance decreased significantly in 5 km competitions and remained unchanged in 10 km and 25 km [23]. One reason for the discrepancy between these results and the outcomes presented in this study could be the observed period of time. While this study implicated swimmers’ competition results over a period of 20 years, others focused on shorter durations [12,23]. Therefore, a possible alteration could have been too slight to be detected when a shorter time period was analyzed.

For annual female finalists, swimming speed improved rather at longer race distances (*i.e.* 800 m freestyle, 1,500 m freestyle, 200 m butterfly, 400 m individual medley). For annual male finalists, swimming speed increased rather at shorter race distances (*i.e.* 100 m freestyle, 200 m freestyle, 100 m butterfly, 100 m breaststroke). Nevill *et al.*[2] investigated the evolution of world records in 100 m, 200 m, 400 m and 800 m freestyle from 1957 to 2006. Both female and male athletes were able to improve their swimming speed in the 1960s and 1970s whereas swimming speeds seemed to plateau between the 1980s and 1990s. This correlates with the findings in Vogt *et al.*[12] and Zingg *et al.*[23]. In both studies, the swimming speed for the fastest female and male athletes remained unchanged across the years. The only exception was stated by Zingg *et al.*[23] where a swimming speed enhancement for men in 10 km competitions was reported. One the one hand, the present findings support these results since swimming speed remained unchanged in roughly half of the analyzed disciplines, distances and races. On the other hand, this study is the first that reports an increase in swimming speed in shorter race distances (*i.e.* 100 m freestyle, 200 m freestyle, 100 m butterfly, 100 m breaststroke) for men and longer race distances (*i.e.* 800 m freestyle, 1,500 m freestyle, 200 m butterfly, 400 m individual medley) for women, respectively. A potential explanation for these disparate findings might be that in contrast to the studies of Vogt *et al.*[12] and Zingg *et al.*[23] enhancements like deeper pools and more effective ‘antiwave’ lane ropes were introduced while the environmental situation remained unchanged in open-water competitions. Regarding the findings from Nevill *et al.*[2], a potential differentiator could be that age as well as multiple participations were not considered. However, in this study all swimming speed results are corrected for multiple participation and age of athletes.

### Changes in sex differences over the years

A further important finding was that the sex difference between the female and male champions at the Olympic Games and FINA World Championships remained stable during the last 20 years. Therefore it seems very likely that the top women will never outperform the top men. However, referring to the finalists at FINA World Championships, the sex difference decreased linearly in 100 m breaststroke and 200 m butterfly whereas it increased non-linearly in 100 m backstroke. Regarding finalists at the Olympic Games the sex difference decreased linearly in 100 m backstroke, 400 m freestyle and 800 m freestyle whereas it increased linearly in 200 m butterfly.

A few authors analyzed whether the swimming speed gap between female and male athletes is narrowing down [2,9]. Nevill *et al.*[2] analyzed the world records from 1957 to 2006 in 100 m, 200 m and 400 m freestyle. The sex difference remained stable and is therefore in line with the findings from the present study. Buhl *et al.*[9] compared medley and freestyle swimming speeds of international swimmers (*i.e.* finalists in the FINA World Championships) between 1994 and 2011 and reported that the sex difference remained unchanged for the 200 m and 400 m medley as well as in the 200 m and 400 m freestyle races. The present findings regarding the decreased sex difference in the Olympic Games and FINA World Championship finalists differ from these findings of Buhl *et al.*[9]. A potential explanation for this disparate finding might be that in the study from Buhl *et al.*[9] a linear regression and one-way analysis of variance was used whereas in this study a multi-level regression analysis with correction for multiple participations and age of athletes was applied.

This study has shown that sex difference decreased with increasing race distance. Different research studies on sex difference evolution with increasing race distance have been conducted over the last decades [7,9-11]. Tanaka and Seals [7] reported a decrease in sex difference with increasing race distance between 1991 and 1995. In detail, for freestyle swimmers the sex difference in swimming speed decreased from 19 ± 1% for 50 m to 11 ± 1% for 1,500 m. Buhl *et al.*[9] compared medley and freestyle swimming speeds for national (*i.e.* top ten elite Swiss athletes) and international swimmers (*i.e.* top eight FINA World Championship athletes) between 1994 and 2011. These authors reported that the sex difference for national and international athletes in 400 m medley as well as freestyle was lower compared to the 200 m disciplines. Wolfrum *et al.*[10] focused on national and international breaststroke and freestyle disciplines and reported a decrease sex difference with increasing race distance. Relating to sex difference, Rüst *et al.*[11] also reported a decrease in sex difference with increasing race distance from 50 m to 800 m amongst Swiss elite freestyle swimmers ranked on the Swiss high score list between 2006 and 2010. However, they reported that for 1,500 m freestyle, the sex difference increased compared to 800 m freestyle. A potential explanation for this disparate finding might be that the study was based on Swiss elite freestyle swimmers only. Also the relatively short time period of about 5 years could be the differentiating factor. All these studies considered only a short period of time, but presented a gap between female and male swimming speeds, which narrows with increasing race distance. With this present study it was possible to reveal that this observation also applies when swimming speeds from over two decades are taken into account.

It seems that women have an advantage with increasing race distance and may even outperform men in long-endurance swimming competitions. The natural difference in body fat percentage might explain the decrease of sex differences with increasing race distance [14,24,25]. A similar explanation was provided by Etter *et al.*[26] and Knechtle *et al.*[27] reporting that the higher body fat percentage of female athletes is an advantage in open-water competitions due to better isolation against the cold water. The lower skeletal muscle mass of female athletes compared to their male counterparts was argued to be another explaining factor of the lower sex difference with increasing race distance [28]. Already in 2010, Knechtle *et al.*[29] reported that male ultra-endurance athletes have a higher skeletal muscle mass than female ultra-endurance athletes. Knechtle *et al.* investigated the skeletal muscle mass of female and male Ironman triathletes [29] and ultra-runners [30]. They reported that male triathletes had an approximately 46% higher skeletal muscle mass compared to their female counterparts. In numbers, male and female triathletes exhibited approximately 41 kg and 28 kg of skeletal muscle mass, respectively [29]. However, male ultra-runners possess with 38 kg an approximately 38% higher skeletal muscle mass than females, which feature 27.4 kg of skeletal muscle mass [30]. Overall, it seems that male athletes have an advantage regarding skeletal muscle mass. Nevertheless, Weitkunat *et al.*[31] showed that male open-water ultra-swimmers experience significant reduction in body mass and skeletal muscle mass. Female open-water ultra-swimmers do not seem to be a subject to this effect, as they show no significant changes with regard to these variables. Another aspect was investigated by Knechtle *et al.*[32-34], whereby these authors analyzed if anthropometric characteristics such as body mass, body height and length of arms have an influence on the overall swimming speed in open-water ultra-distance swimming. They found out that these characteristics did not relate to open-water ultra-distance swimming speed, except body mass index to male swimming speed. Lepers [25] and Lepers and Maffiuletti [35] reported that the underwater torque for female athletes is lower compared to male athletes. On the other hand, female athletes outperform their male colleagues in terms of mechanical efficiency.

Most of these physical factors may explain the development of the gap between female and male swimming speed. However, psychological factors that were not taken into account so far also have a high influence on swimming speed and outcome. Therefore, more studies that focus not only on physical conditions but also on an athlete’s mental status would be needed to fully understand sex differences in elite athletes.

### Limitations

Our study is limited since potential prediction variables such as psychological skills and emotional competencies [6] as well as more efficient training processes based on better training control [5] were not taken into account. Also the improvement in pool design and ‘antiwave lane’ ropes [5] as well as the new drag-reducing swimsuits [4] have not been considered. These variables, environmental as well as material improvements might have influenced race outcomes.

## Conclusions

To summarize, the swimming speed for the overall top eight finalists at FINA World Championships and the Olympic Games increased linearly in all disciplines and distances. Considering men, the top annual swimmers improved it rather at shorter race distances (*i.e.* 100 m freestyle, 200 m freestyle, 100 m butterfly, 100 m breaststroke), whereas the top annual female swimmers improved their swimming speed rather at longer race distances (*i.e.* 800 m freestyle, 1,500 m freestyle, 200 m butterfly, 400 m individual medley). The sex difference remained unchanged in Olympic and FINA World Champions between 1992 and 2013. However, the finalists and champions at the Olympic Games and FINA World Championships decreased the sex difference with increasing race distance. Further studies would be needed to examine how psychological skills, emotional competencies, more efficient training processes based on better training control, improved pool design and ‘antiwave lane’ ropes as well as the new drag-reducing swimsuits influence swimming speed and sex difference. Nevertheless, it seems unlikely that female elite athletes will overtop their male counterparts in any of the indoor swimming disciplines held at FINA World Championships and the Olympic Games.

## Competing interests

The authors declare that they have no competing interest.

## Authors’ contributions

SW collected the data and drafted the manuscript, CR performed the statistical analyses, TR participated in the design of the study, BK helped in collecting the data, in interpretation of the results and in drafting the manuscript. All authors read and approved the final manuscript.

## Pre-publication history

The pre-publication history for this paper can be accessed here:

http://www.biomedcentral.com/2052-1847/6/25/prepub
